# A Competitive Assay Based on Dual-Mode Au@Pt-DNA Biosensors for On-Site Sensitive Determination of Carbendazim Fungicide in Agricultural Products

**DOI:** 10.3389/fnut.2022.820150

**Published:** 2022-02-07

**Authors:** Ge Chen, Rongqi Zhai, Guangyang Liu, Xiaodong Huang, Kaige Zhang, Xiaomin Xu, Lingyun Li, Yanguo Zhang, Jing Wang, Maojun Jin, Donghui Xu, A. M. Abd El-Aty

**Affiliations:** ^1^Key Laboratory of Vegetables Quality and Safety Control, Laboratory of Quality and Safety Risk Assessment for Vegetable Products, Ministry of Agriculture and Rural Affairs, Institute of Vegetables and Flowers, Chinese Academy of Agricultural Sciences, Beijing, China; ^2^Key Laboratory of Agro-Product Quality and Safety, Ministry of Agriculture and Rural Affairs, Institute of Quality Standard and Testing Technology for Agro-Products, Chinese Academy of Agricultural Sciences, Beijing, China; ^3^State Key Laboratory of Biobased Material and Green Papermaking, College of Food Science and Engineering, Qilu University of Technology, Shandong Academy of Science, Jinan, China; ^4^Department of Pharmacology, Faculty of Veterinary Medicine, Cairo University, Giza, Egypt; ^5^Department of Medical Pharmacology, Faculty of Medicine, Atatürk University, Erzurum, Turkey

**Keywords:** carbendazim, Au@Pt-DNA, pesticide residue, biosensor, aptamer

## Abstract

Carbendazim (CBZ), a systemic, broad-spectrum benzimidazole fungicide, is widely used to control fungal diseases in agricultural products. Its residues might pose risks to human health and the environment. Therefore, it is warranted to establish a rapid and reliable method for its residual quantification. Herein, we proposed a competitive assay that combined aptamer (DNA) specific recognition and bimetallic nanozyme gold@platinum (Au@Pt) catalysis to trace the CBZ residue. The DNA was labeled onto bimetallic nanozyme Au@Pt surface to produce Au@Pt probes (Au@Pt-DNA). The magnetic Fe_3_O_4_ was functionalized with a complementary strand of DNA (C-DNA) to form Fe_3_O_4_ probes (Fe_3_O_4_-C-DNA). Subsequently, the CBZ and the Fe_3_O_4_ probes competitively react with Au@Pt probes to form two Au@Pt-DNA biosensors (Au@Pt-ssDNA-CBZ and Au@Pt-dsDNA-Fe_3_O_4_). The Au@Pt-ssDNA-CBZ biosensor was designed for qualitative analysis through a naked-eye visualization strategy in the presence of CBZ. Meanwhile, Au@Pt-dsDNA-Fe_3_O_4_ biosensor was developed to quantitatively analyze CBZ using a multifunctional microplate reader. A competitive assay based on the dual-mode Au@Pt-DNA biosensors was established for onsite sensitive determination of CBZ. The limit of detection (LOD) and recoveries of the developed assay were 0.038 ng/mg and 71.88-110.11%, with relative standard deviations (RSDs) ranging between 3.15 and 10.91%. The assay demonstrated a good correlation with data acquired from liquid chromatography coupled with mass spectrometry/mass spectrometry analysis. In summary, the proposed competitive assay based on dual-mode Au@Pt-DNA biosensors might have a great potential for onsite sensitive detection of pesticides in agro-products.

## Introduction

Carbendazim (CBZ) is a broad-spectrum benzimidazole fungicide used to protect a wide variety of crops against fungal disease, thus producing high-quality crops with optimal yields (1, 2). CBZ, which interferes with DNA biosynthesis during fungal cell division, was defined as a major agrochemical pollutant, hazardous to humans and the environment. Because of its long half-life and severe toxicity, residues might threaten safe consumption and negatively influence food quality (3). There is an urgent need to develop analytical methods to determine residual trace levels of CBZ in agro-products to protect public health. To date, a variety of classical quantitive analyses, including liquid chromatography coupled with mass spectrometry (LC-MS) (4) and gas chromatography coupled with tandem mass spectrometry (GC-MS/MS) (5), have been routinely used to monitor CBZ residues in the agricultural products. Although these analytical methods provide high stability and accuracy, preparation steps are laborious, time-consuming, requiring professional operators and expensive instruments (2), restricting their applications. Therefore, a sensitive, rapid, and simple analytical method is needed to detect CBZ. Currently, the rapid immunoassay for detecting CBZ with high sensitivity and specificity (6) overcomes the pitfalls of the traditional analytical methods (7). However, the antibodies are prone to degradation and denaturation during field applications (8), resulting in difficultly in specifically recognizing the target (9). Moreover, antibodies are more complicated, and the manufacturing process is costly and time consuming (10). Furthermore, haptens synthesized from the CBZ analogs can attach to the appropriate functional groups, enabling the conjugation with the protein (11). Hence, a stable, specific, and cost-effective recognition receptor for CBZ is strongly required to replace the traditional immunoassays.

As molecular recognition elements, aptamers are artificially synthesized single-stranded nucleotide sequences, screened through systematic evolution of ligands by exponential enrichment (SELEX) techniques, and could specifically bind the target analyte (12). Aptamers have attracted significant attention because of their advantages of easy synthesis, low cost, and high affinity (13). Unlike antibodies, aptamers are relatively stable under extreme temperature conditions for a short time (14). Therefore, several platforms of aptamer sensors, such as fluorescence (15), colorimetry (16), and electrochemistry (17), have been developed for the detection of pesticides. To improve the stability and sensitivity of aptamer-based assays, nanomaterials (one of the most interesting sensing materials) are bioconjugated with aptamers for selective and sensitive detection of analytes. The nanomaterials with some enzyme-mimicking characteristics are defined as nanozymes (18). Nanozyme, an emerging alternative to the natural enzyme (19), displayed the following advantages: simple preparation methods, high stability, easy surface modification, and low cost (20). Some enzyme-like bimetallic nanomaterials Au@Pt are of great interest because of their multifunctional and synergistic properties (21). Au@Pt combines good chemical stability of Au with specific catalytic activities of Pt (22). Moreover, the DNA could be firmly bound to the surface of Au@Pt (23). Meanwhile, the recent reports have demonstrated that Au@Pt exhibited high-catalytic properties (24, 25).

**Figure 1:**
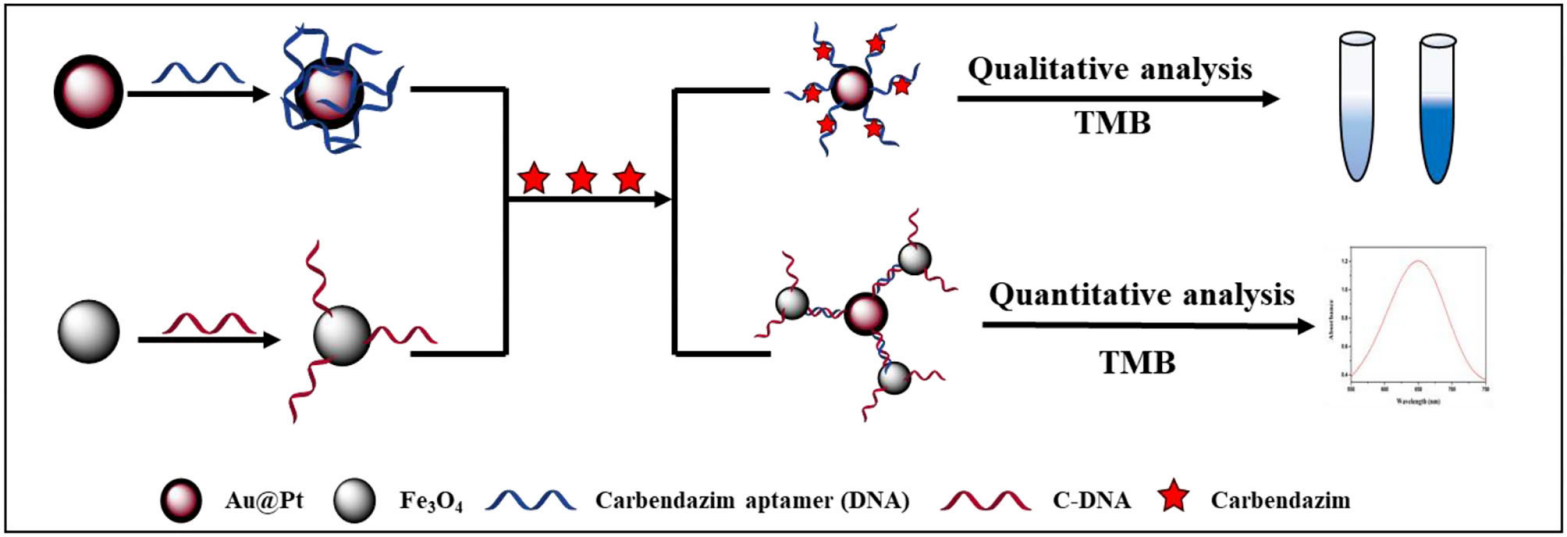
Schematic illustration of a competitive assay based on dual-mode Au@Pt-DNA biosensors for the sensitive determination of CBZ fungicide.

Herein, a competitive assay based on dual-mode Au@Pt-DNA biosensors for onsite sensitive determination of CBZ fungicide is shown in Figure 1. One promising strategy is to reversibly bind target-specific aptamers (DNA) to the Au@Pt surface. The magnetic Fe_3_O_4_ was functionalized with a complementary strand of DNA (cDNA) to form Fe_3_O_4_ probes (Fe_3_O_4_-C-DNA). The CBZ and the Fe_3_O_4_ probes competitively react with DNA-modified nanozyme Au@Pt to form two Au@Pt-DNA biosensors (Au@Pt-ssDNA-CBZ and Au@Pt-dsDNA-Fe_3_O_4_ biosensors, respectively). The aptamers only extend from the Au@Pt surface, specifically in the presence of the CBZ (26), which forms Au@Pt-ssDNA-CBZ biosensor for the qualitative analysis through visualization by naked-eye from light blue to dark blue (19). In addition, Au@Pt-dsDNA-Fe_3_O_4_ biosensor was established for quantitative analysis of CBZ by constructing a calibration curve for trace residual determination of CBZ in agricultural products.

## Materials and Methods

### Materials and Reagents

Gold (Au) chloride hydrate (HAuCl_4_•xH_2_O), chloroplatinic acid (H_2_PtCl_6_•6H_2_O), sodium citrate (C_6_H_5_Na_3_O_7_•2H_2_O, purity > 99%), Fe_3_O_4_ (10 mg/ml), 1-ethyl-3-[3-(dimethylamino) propyl] carbodiimide (EDC), and N-hydroxysuccinimide (NHS) were acquired from Sigma-Aldrich (St. Louis, MO, USA). L-ascorbic acid (C_6_H_8_O_6_, LAA) was procured from Macklin Biochemical Co., Ltd (Shanghai, China). Carboxyl-functionalized magnetic particles (Fe_3_O_4_, 10 mg/ml) were secured from Invitrogen (Grand Island, NY). CBZ standard (C_9_H_9_N_3_O_2_, purity > 99%) was obtained from Dr. Ehrenstorfer GmbH (Augsburg, Germany). Polyethylene glycol 20,000 (PEG 20,000), Tween-20, Tris-EDTA buffer (TE, pH 8.0), and 3,3',5,5'-tetramethylbenzidine (TMB) substrate were purchased from Solarbio (Beijing, China). Primary secondary amine (PSA) and octadecyl (C18) were picked up from Shimadzu (Kyoto, Japan). Anhydrous magnesium sulfate (MgSO_4_), sodium chloride (NaCl), potassium chloride (KCl), disodium phosphate (Na_2_HPO_4_), potassium dihydrogen phosphate (KH_2_PO_4_), and other analytical grade reagents were supplied by Sinopharm Chemical Reagent Co., Ltd. (Beijing, China). Hydrochloric acid (HCl) and nitric acid (HNO_3_) were provided by Beijing Chemical Industry Group Co., Ltd. (Beijing, China). The CBZ aptamer (DNA) and C-DNA (the sequence of DNA and C-DNA designed in Supplementary Table 1) were synthesized by Sangon Co., Ltd. (Shanghai, China). A 96-well micro-plate (Transplant, flat bottom) was supplied by Costar, Inc. (Kennebunk, ME, USA). The HPLC-grade acetonitrile (ACN) and methanol (MeOH) were obtained from Thermo Fisher Scientific (Pittsburgh, PA, USA). Ultrapure water was purified by a Milli-QRC purification system (Millipore, Bedford, MA, USA). Aqua regia is a mixture of HCl and HNO_3_ (HCl/HNO_3_ = 3/1). The 0.01 M phosphate-buffered saline (PBS buffer, pH 7.4) consists of 0.2 g KCl, 0.27 g KH_2_PO_4_, 8 g NaCl, and 1.14 g Na_2_HPO_4_. Washing buffer (PBST: PBS buffer containing 0.05% Tween-20) was prepared for washing micro-plates.

### Preparation of Au@Pt Nanomaterials

The Au@Pt nanomaterials were prepared by the seed-mediated growth method (27). All the glassware was thoroughly soaked in aqua regia and rinsed with ultrapure water. At first, the seeds of gold nanoparticles (AuNPs) were prepared with mirror modifications (28). AuNPs were synthesized by a chemical reduction method where HAuCl_4_ was used as a precursor, and trisodium citrate was used as a reducing agent and stabilizing agent (29, 30). One milliliter HAuCl_4_ (10%, *w/v*) aqueous solution and 99 ml distilled water were added to a round-bottomed flask. After that, 10 ml of trisodium citrate (38.8 mM) solution was quickly added when the aforementioned mixture heated to boiling under vigorous stirring and refluxing using a magnetic stirring heater (Zhengzhou, China). The color of the solution was changed from yellow to wine red, and the mixture was left to stir for another 20 min.

Second, Au@Pt was synthesized using AuNPs (30 ml) as seeds as follows: H_2_PtCl_6_ (1.0 mM, 10 ml) solution and LAA (5 mM, 10 ml) were added to the AuNPs solution and heated to boiling until the color changes from wind red to brown-red. Then, the aforementioned aqueous solution was left to stir for another 30 min to ensure a comprehensive reduction of H_2_PtCl_6_. The synthetic Au@Pt was cooled to room temperature, stored in the dark at 4°C, and then filtered through a cellulose nitrate filter.

### Construction of Au@Pt Probe

Sangon Biotech Co., Ltd. synthesized the aptamer (DNA) of CBZ. The aptamer (DNA) sequence of CBZ was acquired from literature (31). Before using the aptamer (DNA) of CBZ (35.08 μg), it should be activated. At first, 18 μl TE buffer was added into the microcentrifuge tube with the aptamer (DNA) of CBZ to dissolve, and 18 μl TCEP solution was then added to reduce the disulphide bonds to single sulfhydryl groups. Subsequently, the DNA solution was shaken for 3 h under 200 rpm. After that, the activated DNA reacts with Au@Pt solution to form an Au@Pt probe (Au@Pt-DNA). The 30% PEG and 0.1 M PBS were selected to stabilize the Au@Pt probe to achieve 0.5% PEG and 0.01 M PBS in the final solution and avoid the aggregation of the Au@Pt probe (Au@Pt-DNA). Afterward, unbound aptamer of CBZ (DNA) was removed by centrifugation at 10,000 rpm for 30 min. The Au@Pt probe was kept refrigerated at 4°C for further analysis.

### Construction of Fe_3_O_4_ Probe

At first, the C-DNA (33.26 μg) should be activated before conjugation with Fe_3_O_4_. The TE buffer (pH 8.5, 18 μl) was added to the C-DNA. Next, the NHS (10 mg/ml, 100 μl) and EDC (10 mg/ml, 100 μl) were selected to activate the carboxyl group of Fe_3_O_4_ (10 μl, 10 mg/ml) with gentle shaking for 30 min. The Fe_3_O_4_ was magnetically separated and washed three times with MES buffer (15 mM) for 1 min. The washed Fe_3_O_4_ was resuspended in PBS buffer (pH 7.4, 0.1 mol/L). After that, the activated C-DNA was added to Fe_3_O_4_ solution to construct Fe_3_O_4_ probe (Fe_3_O_4_-C-DNA) for 18 h at 4°C. The Fe_3_O_4_ probe was washed three times with PBS buffer to remove the unconjugated C-DNA. Finally, the Fe_3_O_4_ probe was resuspended in 500 μl PBS (pH 7.4, 0.1 mol/L) and stored at 4°C for further use.

### A Competitive Assay Based on Au@Pt-DsDNA-Fe_3_O_4_ Biosensor

In general, the quantitative analysis of CBZ was carried out as follows: at first, 150 μl Au@Pt-DNA (0.3 nmol/L) was added to a 0.5-ml microcentrifuge tube. Then, different concentrations of CBZ standard were prepared by PBS solution containing 10% methanol or supernatant of actual samples extract in advance. Afterward, 60 μl CBZ was added to Au@Pt-DNA solution, followed by adding a 60 μl Fe_3_O_4_ probe (diluted by probe buffer, 0.1 mg/L) to construct a competitive assay at room temperature. The aforementioned mixture was washed three times, followed by separation under a magnetic field for 1 min. After that, the Au@Pt-dsDNA-Fe_3_O_4_ mixture was resuspended in PBS buffer to catalyze TMB for the trace detection of CBZ using a multifunctional microplate reader (Salzburg, Austria).

### Sample Preparation

The leeks and rice samples were obtained from the Shangdong vegetable production field to evaluate the feasibility of the developed assay. Blank samples (free from CBZ) were used for creating a calibration curve and recovery experiments. The leeks and rice samples were pretreated with QuEChERS (quick, easy, cheap, effective, rugged, and safe) method designed by the Anastassiades team (32) with slight modifications. Standard concentrations (10, 50, and 100 μg·kg^−1^) were used as spiking levels to homogenized samples (10 g for leeks, 5 g for rice) in 50-ml centrifuge tubes. Afterward, the spiked samples were allowed to equilibrate at room temperature for 4 h. Subsequently, 10 ml acetonitrile was added, followed by a 2 min vortex mix. Next, 2 g anhydrous MgSO_4_ and 1 g NaCl were added for dehydration and stratification (vigorous shaking for 1 min). The supernatant was transferred into a 10-ml plastic tube, vortexed for another 1 min, and centrifuged at 5,000 rpm (at 4°C) for 5 min. Subsequently, 5 ml supernatant was purified using a purification cartridge (52 mg PSA, 52 mg C18, and 26 mg GCB), vortexed again for 1 min, and then centrifuged at 10,000 rpm (at 4°C) for 5 min. Finally, the supernatant was filtered through a 0.22-μm filter (Jinteng, China) for competitive assay based on Au@Pt-dsDNA-Fe_3_O_4_ biosensor and LC-MS/MS analysis.

## Results

### Characterization of Au@Pt and Au@Pt Probe

**Figure 2:**
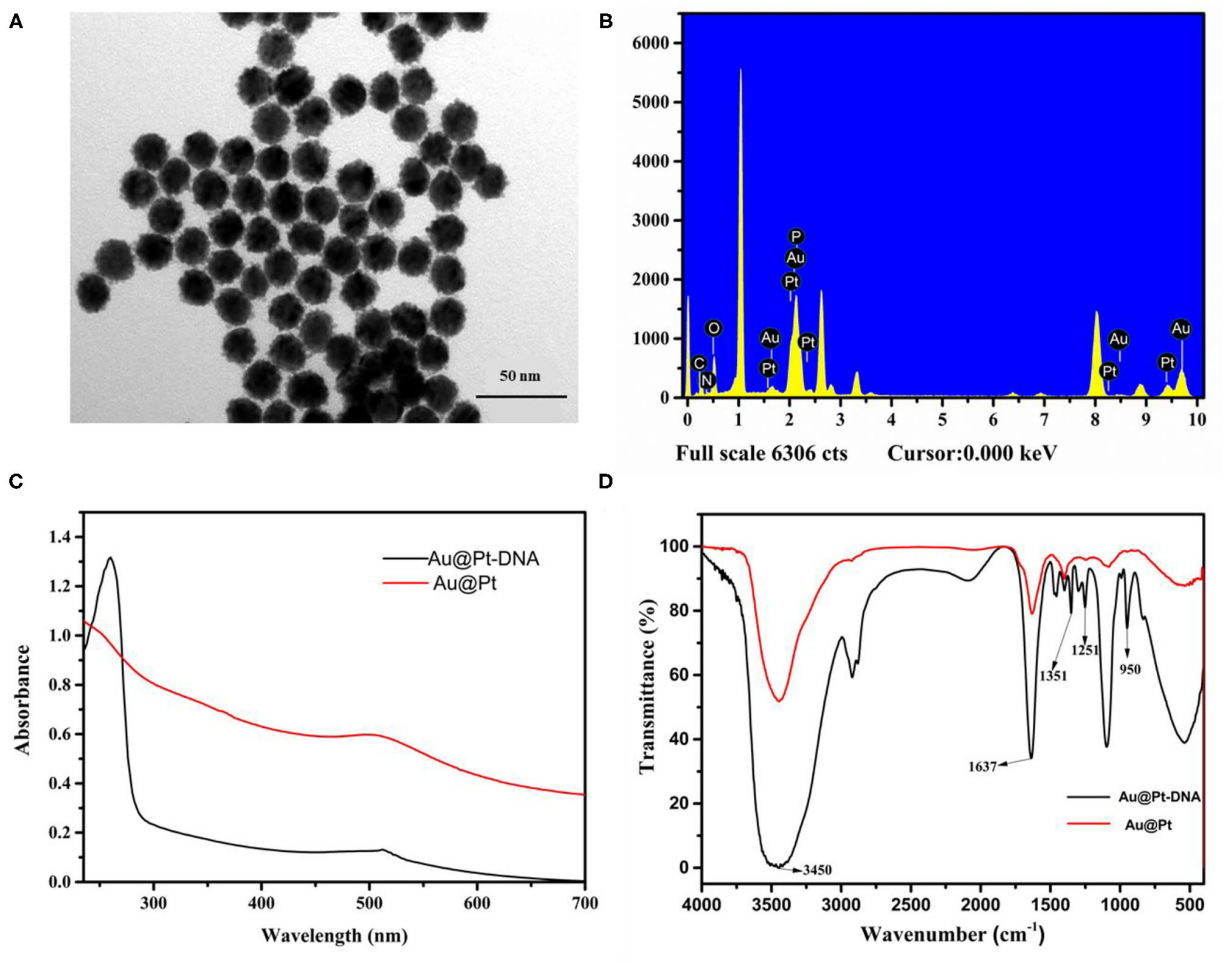
Characterization of Au@Pt and Au@Pt probes. **(A)** TEM of Au@Pt; **(B)** EDS of Au@Pt probe; **(C)** UV-Vis spectra of Au@Pt and Au@Pt probes; and **(D)** FTIR spectra of Au@Pt and Au@Pt probes.

Using Au as seeds, a thin Pt layer was deposited on the surface of the Au core to achieve Au@Pt. The morphology of Au@Pt nanoparticles was characterized by transmission electron microscopy (TEM). As shown in Figure 2A, the Au@Pt showed uniform spherical shapes with an average particle size of 20 nm. Compared with smooth Au (as shown in Supplementary Figure 1A). The particle size of Au@Pt was increased by ~5 nm indicating the existence of smooth AuNPs surface of a 5 nm layer Pt. The TEM images of Au@Pt demonstrated that the Au@Pt core-shell structure was successfully prepared. Then, the Au@Pt and Au@Pt probes were carefully characterized by energy dispersive spectrometer (EDS). As presented in Figure 2B, the EDS of Au@Pt probe has shown that *P* element signal except for Au and Pt, which are from Au@Pt nanoparticles (as depicted in Supplementary Figure 1B). Therefore, the *P* characteristic element can be inferred from the DNA. The EDS (Hitachi Co., Ltd., Tokyo, Japan) of Au@Pt and Au@Pt probes indicate that the DNA was successfully modified on the Au@Pt surface. To further confirm the successful preparation of the Au@Pt probe, the UV–vis spectra (Shimadzu, Japan) were used to verify the synthesized Au@Pt probe. The UV–vis spectra of Au@Pt (red line) have a maximum absorbance at 508 nm. Compared with Au@Pt, a prominent characteristic absorbance peak near 260 nm was noticed in UV–vis spectroscopy of Au@Pt probe (black line), as presented in Figure 2C. As reported, the maximum absorption peak of DNA occurs at 260 nm (23), denoting that the Au@Pt surface was conjugated with DNA. In addition, the Au@Pt probe was characterized by FT-IR (Pittsburgh, USA), as depicted in Figure 2D. Peaks around 3,450 and 1,637 cm^−1^ are derived from the O–H stretching, representing the H-O-H bending vibration of water. The characteristic peaks of 1,351, 1,251, and 950 cm^−1^ were observed except for 3,450 and 1,637 cm^−1^ in the Fourier-transform infrared spectroscopy (FTIR) spectra of the Au@Pt probe. These characteristic peaks, corresponding to C=C, C-N, and C-H, respectively, can be inferred from bases of DNA. These results denote that the DNA was successfully modified on the surface of Au@Pt.

### Optimization of the Experimental Conditions

**Figure 3:**
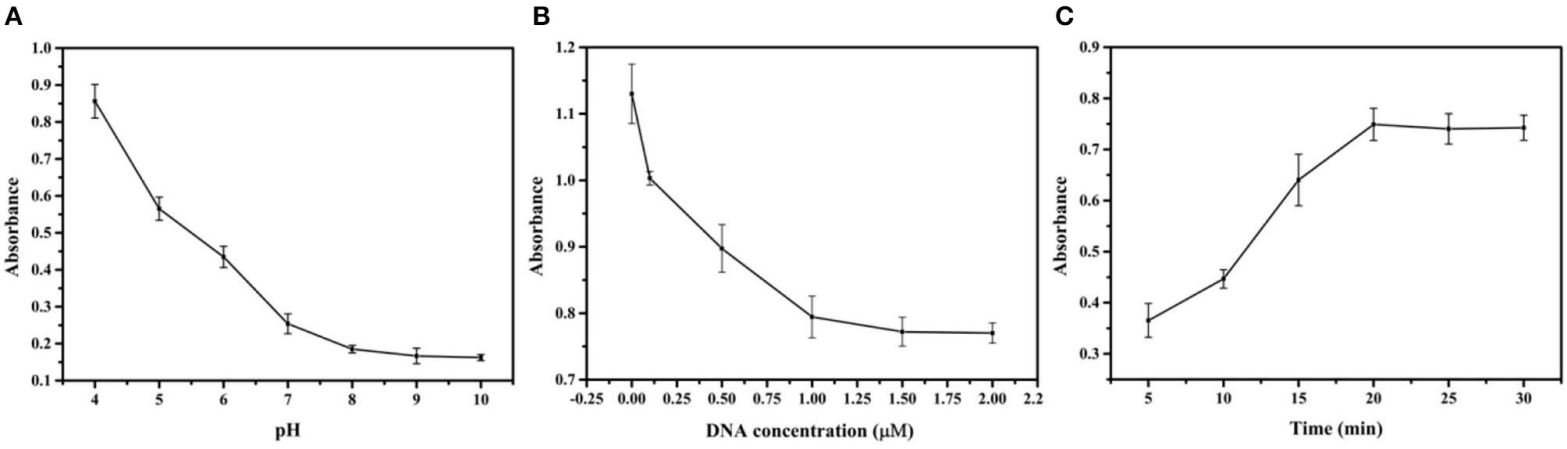
Optimization of the experimental conditions. **(A)** pH; **(B)** DNA concentration; and **(C)** reaction time.

This section investigated the influence of important factors, such as pH value, DNA concentration, and reaction time, to achieve the optimal experimental conditions for CBZ detection. The pH plays a crucial role in the preparation of the Au@Pt probe. A series of pH ranges (4.0, 5.0, 6.0, 7.0, 8.0, 9.0, and 10.0) was set to estimate the optimal value for forming the Au@Pt probe. The catalytic efficiency of the Au@Pt probe was decreased with increasing the pH value to 8.0 (as depicted in Figure 3A). The DNA is easily degraded under acidic conditions (low pH value). The Au@Pt probe catalytic efficiency was increased because more catalytic active sites were exposed with a low concentration of DNA modified on the surface of Au@Pt nanomaterials. Thus, the optimal pH value was set at 8.0 for this assay. The sensitivity of the competitive assay based on Au@Pt-dsDNA-Fe_3_O_4_ biosensor depends on the DNA concentrations modified on the surface of Au@Pt. Then, the DNA concentration was carefully optimized as well. Different DNA concentrations (0, 0.1, 0.5, 1.0, 1.5, and 2.0 μM) were designed to prepare the Au@Pt probe. Like the influence of pH, the catalytic efficiency of the Au@Pt probe was reduced with a high concentration of DNA, as can be displayed from Figure 3B, reaching maximum absorbance at 1.0 μM. Notably, this evidence demonstrated that the optimal concentration of DNA was 1.0 μM in this assay. It is essential to optimize the reaction time between CBZ and Au@Pt probe to detect CBZ rapidly. In turn, a set of reaction times (5, 10, 15, 20, 25, and 30 min) were designed to achieve the optimal reaction time. As time increased, the catalytic efficiency of the Au@Pt probe was improved, reaching the maximum absorbance at 20 min (as shown in Figure 3C). Thus, 20 min was identified as the optimized reaction time for the following experiments.

### Qualitative Analysis

**Figure 4:**
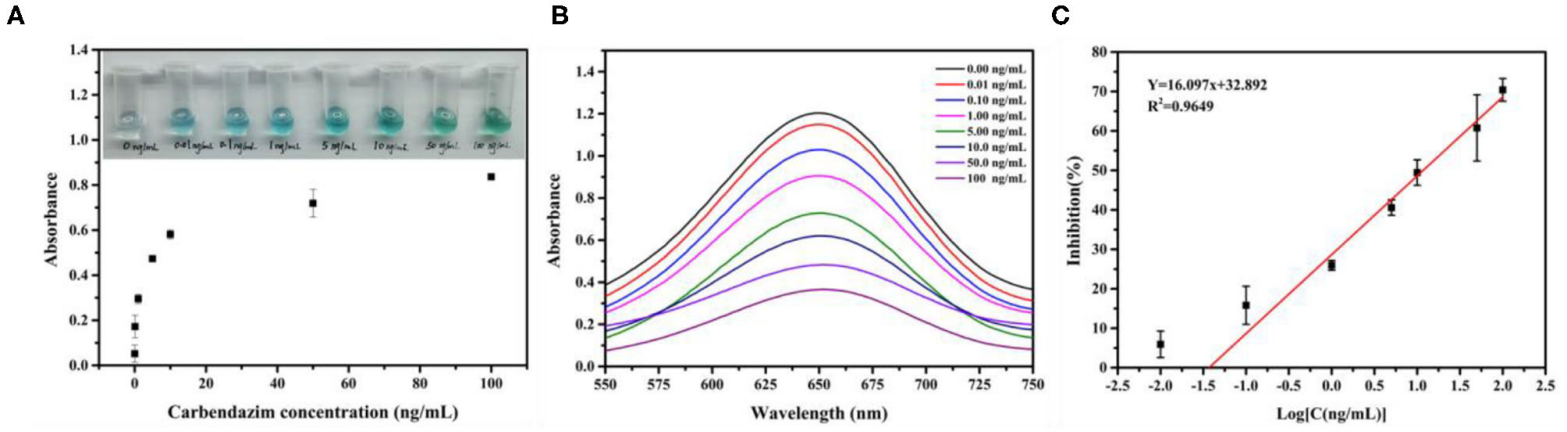
Construction of the calibration curve. **(A)** UV–vis spectra absorbance for various concentrations of carbendazim (CBZ); **(B)** the absorbance scan with CBZ concentration (0.00–100 ng/ml); and **(C)** calibration curve of a competitive assay based on Au@Pt-dsDNA-Fe_3_O_4_ biosensor (*n* = 3).

The CBZ and the Fe_3_O_4_ probe competitively react with DNA-modified nanomaterials Au@Pt to form Au@Pt-ssDNA-CBZ biosensor and Au@Pt-dsDNA-Fe_3_O_4_ biosensor, respectively. The Au@Pt-ssDNA-CBZ biosensor solution color dramatically changed from light blue to dark blue with Au@Pt-ssDNA-CBZ biosensor to catalyze TMB when the concentration of CBZ increased from 0 to 100 ng/ml (as shown in Figure 4A). The catalytic efficiency of the Au@Pt-ssDNA-CBZ biosensor was improved by increasing the CBZ concentration. The Au@Pt-ssDNA-CBZ biosensor catalytic efficiency was increased because more catalytic active sites were exposed in the presence of CBZ, which was bonded to the DNA on the surface of Au@Pt nanomaterials. Hence, the designed Au@Pt-ssDNA-CBZ biosensor could use as a qualitative analysis biosensor for on-site pesticide monitoring in the agro-products.

### Quantitative Analysis (Calibration Curves)

The calibration curve was established based on Au@Pt-dsDNA-Fe_3_O_4_ biosensor catalysis in the competitive assay. The absorbance intensity decreased as the CBZ concentration increased in this study, as depicted in Figure 4B. It displayed a maximum absorbance at 650 nm. The calibration curve of the competitive assay based on Au@Pt-dsDNA-Fe_3_O_4_ biosensor for detection of CBZ pesticide was established under the optimal condition (Figure 4C). The calibration regression equation of curve (*Y* = 16.097x + 32.892) was achieved with a wide linear range. Y represented the inhibition rate (%) and the logarithmic (Log) concentration of CBZ as the X abscissa axis. A low-detection limit (0.038 ng/mg) was acquired with a good linear relationship (*R*^2^ = 0.9649). The RSDs ranged from 1.56 to 10.22%. The LOD of this assay was lower than the maximum residue limit (MRL) of CBZ enacted by China (GB 2763–2021) in various agro-products.

### Assay Evaluation

The method was validated in terms of accuracy, precision, and LODs. The accuracy (recoveries of the proposed assay), precision (RSDs of the proposed assay), LODs, and correlation coefficients (*R*^2^ of regression equation) were used to evaluate the reliability, applicability, and sensitivity of the assay. To determine the validation parameters, three concentrations (10, 50, and 100 ng/mg) of CBZ standard solutions were chosen. For leek samples, the average recoveries ranged from 71.88 to 110.11% with RSDs (3.93–10.91%), and a low LOD (0.044 ng/mg) and IC_50_ (7.56 ng/mg) were acquired in the competitive assay. Like leeks', average recoveries of rice, RSDs, LOD, and IC_50_ were 89.86–107.45%, 4.21–6.52%, 0.041 ng/mg and 6.08 ng/mg, respectively (Table 1). The regression equations were *Y* = 17.905x + 34.267 and *Y* = 18.718x + 36.535 for leeks and rice, respectively. Hence, good correlations (*R*^2^ 0.9613 for leeks and 0.9453 for rice) were gained using the competitive assay (Table 1, Supplementary Figure 3). Therefore, the Au@Pt-dsDNA-Fe_3_O_4_ biosensor of this competitive assay holds the potential as a sensitive and reliable assay for residual trace detection of pesticides.

**Table 1:** Calibration curve of carbendazim (CBZ) in field incurred samples (*n* = 3).

**Samples**	**Spiked concentration** **(ng/mg)**	**Recovery** **(%)**	**RSD (%)**	**IC_**50**_** (**ng/mg)**	**LOD** **(ng/mg)**	***R** ^**2**^*
Leek	10	71.88 ± 0.04	10.91	7.56	0.044	0.9613
	50	96.40 ± 0.01	3.93			
	100	110.11 ± 0.02	6.76			
Rice	10	91.62 ± 0.03	6.52	6.08	0.041	0.9453
	50	89.86 ± 0.01	4.21			
	100	107.45 ± 0.01	3.16			

*C_50_: Represents the concentration at which a substance exerts half of its maximal inhibitory effect*.

### Specificity

**Figure 5:**
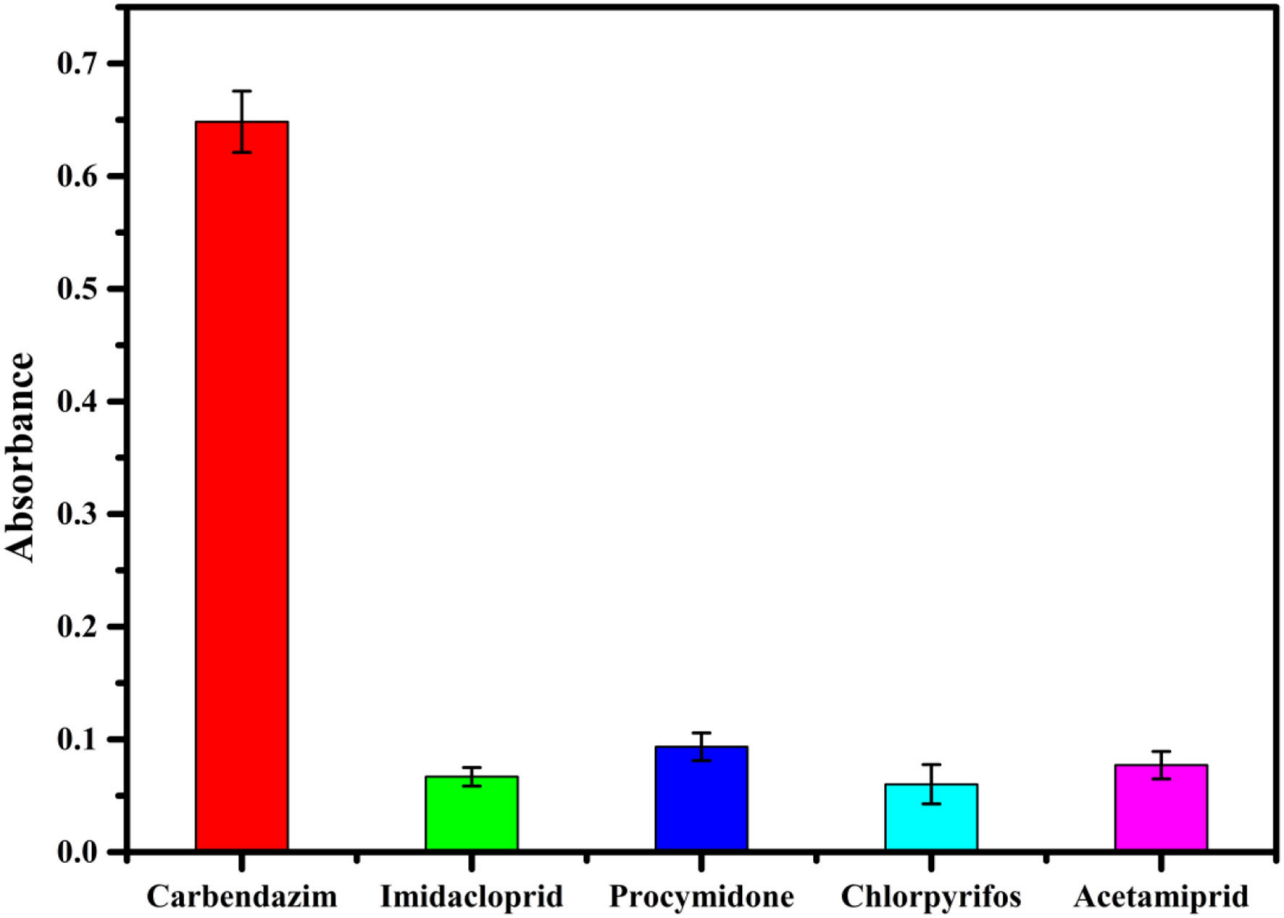
Specificity between CBZ and other unrelated pesticides.

Specificity, recognizing the target molecule, is essential in the performance evaluation (33). The accuracy of the assay method is mainly based on the specificity of the CBZ aptamer (2). Several commonly used fungicides and insecticides (100 ng/ml solution of imidacloprid, procymidone, chlorpyrifos, and acetamiprid) were investigated to evaluate the specificity of the proposed competitive assay for detection of CBZ. As can be inferred from Figure 5, the absorbance of the Au@Pt-ssDNA-CBZ biosensor increased only in the presence of CBZ. In contrast, other pesticides caused minor changes in absorbance. Hence, imidacloprid, procymidone, chlorpyrifos, and acetamiprid have a negligible effect on the proposed competitive assay in the presence of CBZ.

### Confirmation Analysis

**Figure 6:**
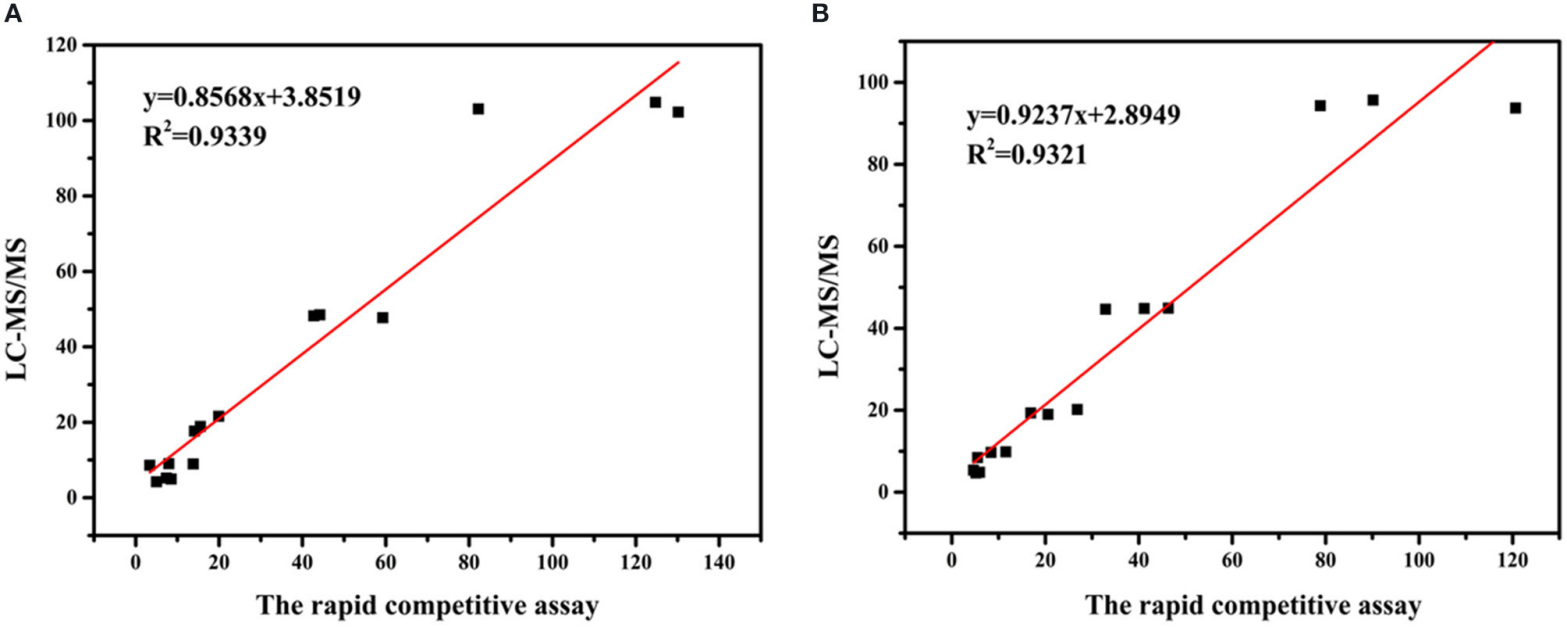
Correlation between the concentrations of CBZ measured by the proposed competitive assay based on Au@Pt-dsDNA-Fe_3_O_4_ mediated biosensor and LC–MS/MS in **(A)** leeks and **(B)** rice.

To further confirm the proposed accuracy of the assay, the blank leeks and rice samples were randomly selected to establish the correlation between the competitive assay and LC-MS/MS method (the parameters of LC-MS/MS shown in Supplementary Table 2). Five concentrations (5, 10, 20, 50, and 100 ng/mg) of CBZ were spiked to blank leek (*n* = 24) and rice (*n* = 24) samples with vigorous shaking. Half of the samples were analyzed by the proposed competitive assay based on Au@Pt-dsDNA-Fe_3_O_4_ biosensor, and the rest were quantified with LC-MS/MS. The correlation coefficients were achieved to assess the association between both methods. The correlation coefficients were 0.9339 and 0.9321 for leek and rice, respectively (Figure 6, Supplementary Table 3). These findings denote that competitive assay has good reliability to satisfy the requirements for pesticide detection in agro-products.

## Discussion

This study proposed dual-mode Au@Pt-DNA biosensors (Au@Pt-ssDNA-CBZ biosensor and Au@Pt-dsDNA-Fe^3^O^4^ biosensor) to analyze CBZ residues agricultural products.

The construction of the Au@Pt probe is the main key factor for Au@Pt-DNA biosensors. The EDS, UV–vis and FT-IR techniques were used to characterize the Au@Pt probe. Compared with Au@Pt nanoparticles (as depicted in Supplementary Figure 1B), the EDS of the Au@Pt probe has shown a *P* element signal (Figure 2B). It has been deduced that the *P* element is derived from DNA. A prominent characteristic absorbance peak (near 260 nm) was noticed in UV–vis spectroscopy of the Au@Pt probe (Figure 2C). As reported, the maximum absorption peak of DNA occurs at 260 nm (23). The characteristic peaks of 1,351, 1,251, and 950 cm^−1^, corresponding to C=C, C-N, and C-H, were observed in the FTIR spectra of the Au@Pt probe, as shown in Figure 2D. These results denote that the Au@Pt probe was successfully prepared.

The pH value, DNA concentration, and reaction time were investigated for establishing the assay based on Au@Pt-DNA biosensors to detect CBZ. The DNA is easily degraded under acidic conditions. The catalytic efficiency of the Au@Pt probe achieved the best results when the pH value reached 8.0 (Figure 3A). Similarly, the concentration of DNA at 1.0 μM, the catalytic efficiency of the Au@Pt probe reached maximum absorbance (Figure 3B). The Au@Pt probe with low DNA concentration achieves poor sensitivity. However, the higher DNA concentration inhibits the Au@Pt probe catalysis efficiency because too much DNA concentration might be covering the catalytic site on the Au@Pt nanozyme.

The Au@Pt-ssDNA-CBZ biosensor solution color dramatically changed from light blue to dark blue with Au@Pt-ssDNA-CBZ biosensor to catalyze TMB when the concentration of CBZ increased from 0 to 100 ng/ml (as shown in Figure 4A). The catalytic efficiency of the Au@Pt-ssDNA-CBZ biosensor improved by increasing the CBZ concentration because more catalytic active sites of Au@Pt were exposed in the presence of CBZ that was bonded to the DNA on the surface of Au@Pt nanomaterials. Hence, the designed Au@Pt-ssDNA-CBZ biosensor could use as a qualitative analysis biosensor for on-site pesticide monitoring in agro-products through naked-eye visualization.

In addition, the Au@Pt-dsDNA-Fe_3_O_4_ biosensor was regarded as quantitate analysis biosensor to establish a sensitive and reliable assay for residual trace detection of pesticides. The regression equation of calibration curve' (*Y* = 16.097x + 32.892, *R*^2^ = 0.9649) was achieved. This assay acquired a low LOD (0.038 ng/mg) with RSDs ranged from 1.56 to 10.22% (Figure 4C). The leeks and rice samples were selected to monitor the residual levels of CBZ using the proposed assay. As shown in Table 1, the mean recoveries (leeks: 71.88–110.11%, rice: 89.86–107.45%) meet the EU guidance document for pesticide residue testing (SANTE/11813/2017). This assay acquired a low LOD (0.038 ng/mg) with RSDs ranging from 1.56 to 10.22%. Therefore, the Au@Pt-dsDNA-Fe_3_O_4_ biosensor of this competitive assay holds the potential as a sensitive and reliable assay for residual trace detection of pesticides. Compared with other assays based on nanomaterials, such as Au/Fe_3_O_4_ (6), UCNPs-MnO_2_ (34), AuNPs (35, 36), Nd_2_O_3_ (37), and MoS_2_/MWCNTs (38) for CBZ detection is compiled in Table 2. As shown, this proposed competitive assay based on dual-mode Au@Pt-DNA biosensors acquires much lower LOD than other nanomaterials. Besides, the LOD of this assay is much lower than the MRL of CBZ set by China in various agro-products.

**Table 2:** Various assays for detection of CBZ based on nanomaterials.

**Nanomaterials**	**Recovery (%)**	**LOD**	**Reference**
Au@Pt	71.88–110.11%	0.037 ng/mg	This work
Au/Fe_3_O_4_	102.4–115.0%	0.44 ng/ml	(6)
UCNPs-MnO_2_	85.58–109.40%	0.05 ng/ml	(34)
AuNPs	94.9–104.8%	2.2 nM	(35)
AuNPs	96.3–111.2%	2.33 nM	(36)
Nd_2_O_3_	-	0.027 mM	(37)
MoS_2_/MWCNTs	89.18–105.56%	7.4 nM	(38)

## Conclusions and Future Perspectives

A competitive assay based on dual-mode Au@Pt-DNA biosensors combined aptamer (DNA) specific recognition property, bimetallic nanomaterials Au@Pt catalysis, and Fe_3_O_4_ magnetic separation was proposed to trace the CBZ residue. The qualitative Au@Pt-ssDNA-CBZ biosensor with aptamer (DNA) specific recognition property monitors pesticides through light blue to dark blue visualization and quantitative Au@Pt-dsDNA-Fe_3_O_4_ biosensor with bimetallic nanomaterials Au@Pt catalysis and Fe_3_O_4_ magnetic separation detect CBZ residue, respectively. Overall, this proposed competitive assay holds the potential as a sensitive and reliable assay for residual trace detection of pesticides and can be used for rapid on-site pesticide monitoring, reducing false-negative results, and improving screening efficiency and sensitivity.

## Data Availability Statement

The original contributions presented in the study are included in the article/Supplementary Material, further inquiries can be directed to the corresponding authors.

## Author Contributions

GC and DX investigated, designed, and wrote the original draft. RZ, GL, and XH investigated and supervised the article. KZ, XX, LL, and YZ visualized and investigated. MJ, JW, and AA were involved in investigation and writing (review and editing) the manuscript. All the authors contributed to the article and approved the submitted version.

## Funding

This study was financially supported by Central Public-interest Scientific Institution Basal Research Fund, Chinese Academy of Agricultural Sciences (IVF-BRF2021020), the Agricultural Science and Technology Innovation Program of CAAS (CAAS-ZDRW202011 and CAAS-TCX2019025-5), the China Agriculture Research System of MOF and MARA (CARS-23-E03), and the National Key Research Development Program of China (2020YFD1000300).

## Conflict of Interest

The authors declare that the research was conducted in the absence of any commercial or financial relationships that could be construed as a potential conflict of interest.

## Publisher's Note

All claims expressed in this article are solely those of the authors and do not necessarily represent those of their affiliated organizations, or those of the publisher, the editors and the reviewers. Any product that may be evaluated in this article, or claim that may be made by its manufacturer, is not guaranteed or endorsed by the publisher.
