# Supplementary material for: A Competitive Assay Based on Dual-Mode Au@Pt-DNA Biosensors for On-Site Sensitive Determination of Carbendazim Fungicide in Agricultural Products
Source: Front Nutr. 2022 Feb 7;9:820150. doi: 10.3389/fnut.2022.820150 (PMC8860170; doi:10.3389/fnut.2022.820150)
Supplement: Supplementary file 1 [file Data_Sheet_1.docx]

**Supplementary Material**

**A** **competitive Assay** **Based on Dual-mode Au@Pt-DNA Biosensors for on-site sensitive Determination of** **Carbendazim Fungicide in Agricultural Products**

**Ge Chen^1*^, Rongqi** **Zhai^1^, Guangyang Liu^1^, Xiaodong Huang^1^, Kaige Zhang^1^, Xiaomin Xu^1^, Lingyun** **Li^1^, Yanguo Zhang^1^, Jing Wang^2^, Maojun Jin^2^, Donghui Xu^1*^, A. M. Abd El-Aty^3,4,5^**

*^1^Institute of Vegetables and Flowers, Chinese Academy of Agricultural Sciences, Key Laboratory of Vegetables Quality and Safety Control, Laboratory of Quality & Safety Risk Assessment for Vegetable Products, Ministry of Agriculture and Rural Affairs, Beijing, China*

*^2^Institute of Quality Standard and Testing Technology for Agro-Products, Chinese Academy of Agricultural Sciences, Key Laboratory of Agro-Product Quality and Safety, Ministry of Agriculture and Rural Affairs, Beijing 100081, PR China*

*^3^State Key Laboratory of Biobased Material and Green Papermaking, College of Food Science and Engineering, Qilu University of Technology, Shandong Academy of Science, Jinan 250353, China*

*^4^Department of Pharmacology, Faculty of Veterinary Medicine, Cairo University, 12211 Giza, Egypt*

*^5^Department of Medical Pharmacology, Faculty of Medicine, Atatürk University, Erzurum, Turkey*

1. **Oligonucleotide sequences**

**TABLE S1** Oligonucleotide sequences of carbendazim aptamer (DNA) and complementary DNA.

| Oligonucleotide | Aptamer (DNA) recognition sequence and complementary DNA sequence |
| --- | --- |
| DNA | GGGCACACAACAACCGATGGTCCAGCCACCCGAATGACCAGCCCACCCGCCACCCCGCG |
| C-DNA | CGCGGGGTGGCGGGTGGGCTGGTCATTCGGGTGGCTCCACCATCGGTTGTTGTGTGCCC |

1. **Calculation of inhibition rate**

$$I\%=\frac{（A_{max}- A_{0}）- (A_{x}- A_{0})}{(A_{max}- A_{0})}\times100$$

where I% represents inhibition rate; A_max_ represents the absorbance of a blank experiment at 650 nm; A_0_ denotes the absorbance of background interference at 650 nm; A_x_ means the biosensor's absorbance in presence carbendazim at 650 nm.

**3. Characterization of Au and Au@Pt**


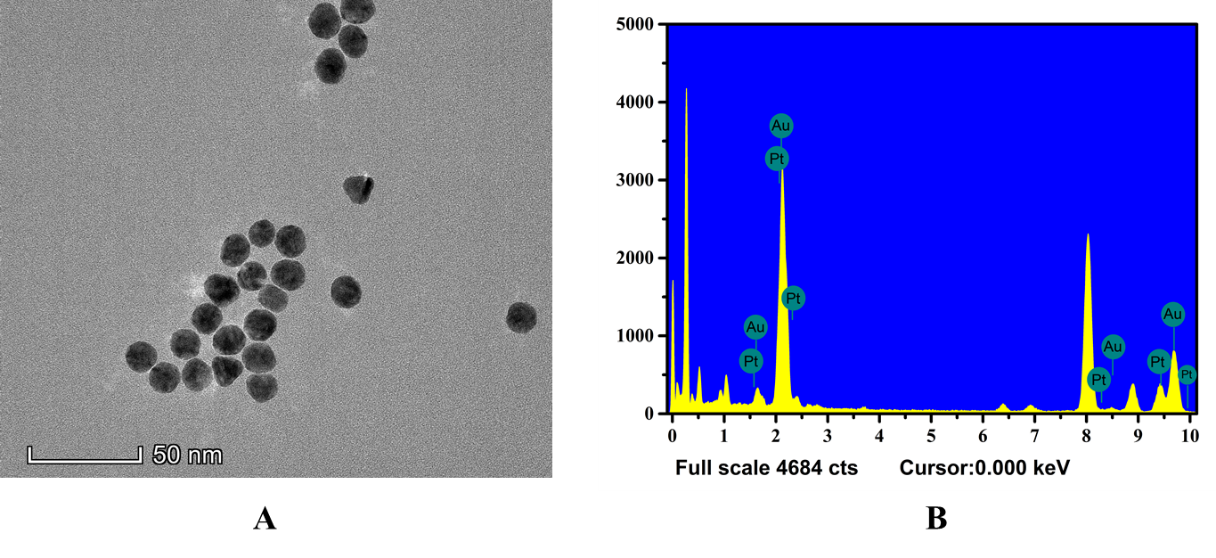


**FIGURE S1**. (A) the TEM of Au; and (B) the EDS of Au@Pt.

**4. Characterization of Fe_3_O_4_ probe**

The characterization of the Fe_3_O_4_ probe was conducted by TEM and FTIR techniques. Like the Au@Pt biosensor, the TEM images of Fe_3_O_4_ are shown in Figure S2. The Au@Pt showed uniform spherical shapes with an average particle of 1 μm. Similar to the TEM images, the Fe_3_O_4_ probe also demonstrated characteristic peaks at 1655 cm^−1^ and 1399 cm^−1^. The distinct peaks (1655 cm^−1^ and 1399 cm^−1^) were inferred from the N-H bond and C=O bond of DNA, respectively. These results denote that the Fe_3_O_4_ probes were successfully synthesized.


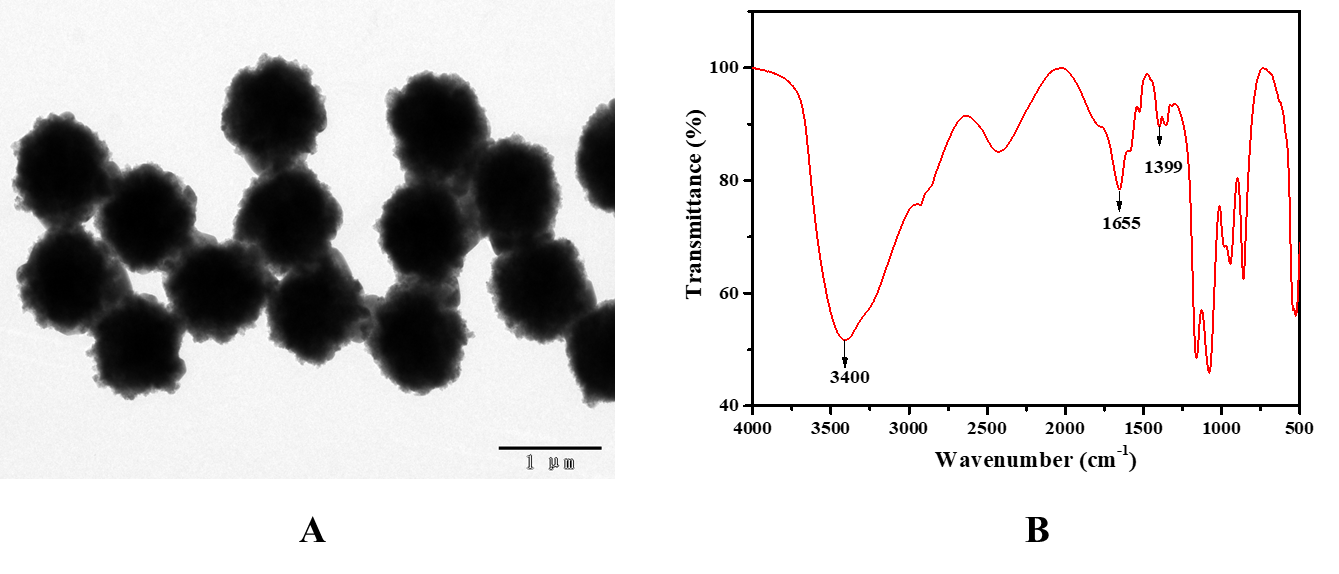


**FIGURE S2**. (A) The TEM of Fe_3_O_4_; and (B) the FTIR spectra of Fe_3_O_4_ probe.


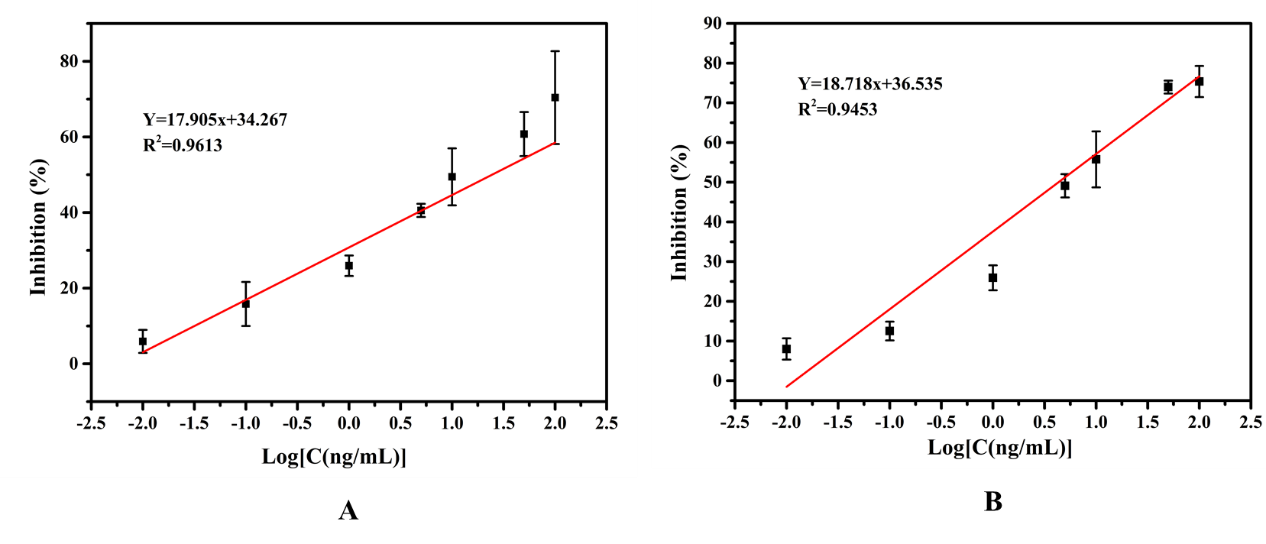


**FIGURE S3**. Calibration curves of carbendazim in field incurred samples. (A) leeks; and (B) rice.

**5. Effect of salt ions on hybridization of DNA**

The hybridization of DNA has directly affects Au@Pt-dsDNA-Fe_3_O_4_ biosensor quantification. A series of concentrations of Na^+^ and Mg^2+^ was set up to investigate the effect on the Au@Pt-dsDNA-Fe_3_O_4_ sensor. As shown in FIGURE S4, Na^+^ could reduce the DNA Debye radius because of the negative charge of the phosphate backbone, and high sodium ion concentrations result in a small Debye radius and low hybridization resistance caused by the negative charge of the phosphate backbone. The higher the concentration of Na^+^, the smaller the Debye radius, resulting in less resistance to DNA hybridization. However, too much Na^+^ can lead to aggregation of the nanozyme Au@Pt and affect the catalytic activity of the Au@Pt. The maximum Na^+^ concentration in the reaction system is 0.13 mol/L in the case of Au@Pt that do not develop into aggregates. Therefore, Na^+^ concentration is 0.13 mol/L in this study. Mg^2+^ is an element required by DNA polymerase. DNA has only 59 bp sequences and relies mainly on base complementary pairing for the hybridization, which does not require polymerase in this study. Thence, Mg^2+^ does not affect this experiment.


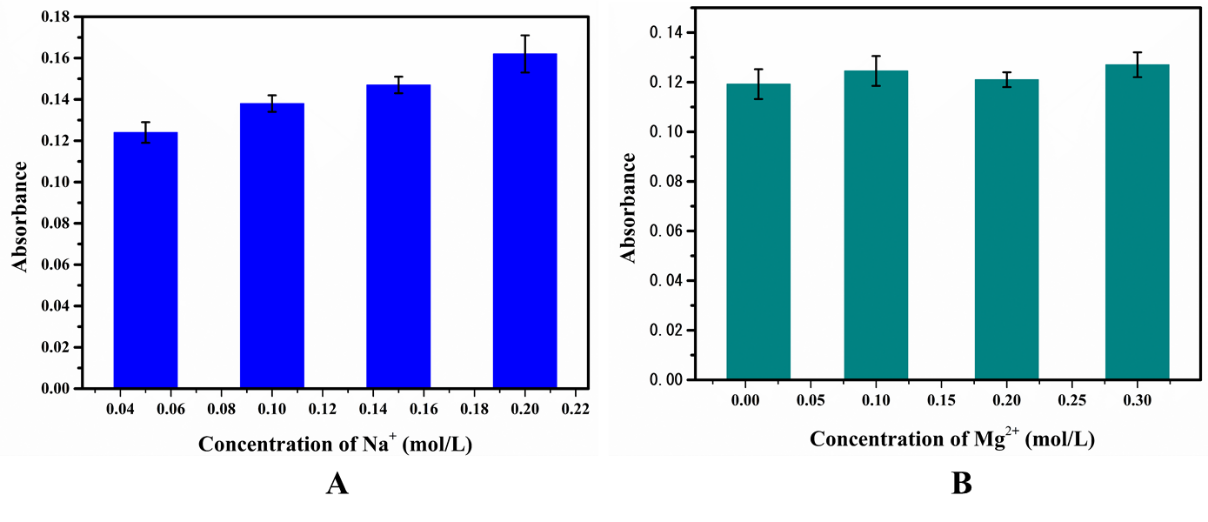


**FIGURE S4**. Effect of salt ions on hybridization of DNA. (A) Na^+^; and (B) Mg^2+^.

**6. LC-MS/MS conditions**

The confirmation analysis was carried out with LC-MS-8050 and Nexera LC system (Shimadzu, Kyoto, Japan).

Phenomenex-C18 (50 mm×3 mm, 2.6 μm, Kinetex (Boston, USA);

The injection volume: 10 μL

The mobile phase A: 1mmol/L ammonium acetate solution

Mobile phase B: methanol

The flow rate: 0.3 mL/min

Column temperature: 40 ℃

Retention time: 10.855 min

Quantitative ion pairs：192/160.1

Qualitative ion pairs：192/132.2

**TABLE S2**. Gradient elution conditions.

| Time (min) | Mobile phase A | Mobile phase B | |
| --- | --- | --- | --- |
| 0 | 80% | 20% | |
| 8 | 5% | 95% | |
| 12 | 80% | 20% | |
|  |  |  |  |

**TABLE S3**. Confirmation statistical analysis (n=3).

| CBZ concentration (ng/mg) | Leek | | Rice | |
| --- | --- | --- | --- | --- |
|  | Au@Pt-DNA biosensor | LC-MS/MS | Au@Pt-DNA biosensor | LC-MS/MS |
| 5 | 7.36 | 5.24 | 5.16 | 4.67 |
|  | 4.99 | 4.24 | 4.64 | 5.37 |
|  | 8.51 | 4.97 | 5.94 | 4.86 |
| 10 | 3.39 | 8.60 | 11.54 | 9.85 |
|  | 13.79 | 8.95 | 5.49 | 8.46 |
|  | 7.94 | 9.03 | 8.40 | 9.72 |
| 20 | 15.51 | 18.90 | 16.88 | 19.34 |
|  | 19.93 | 21.57 | 26.84 | 20.16 |
|  | 14.12 | 17.65 | 20.57 | 18.97 |
| 50 | 44.19 | 48.51 | 32.90 | 44.67 |
|  | 59.29 | 47.72 | 41.16 | 44.84 |
|  | 42.74 | 48.24 | 46.31 | 44.91 |
| 100 | 124.71 | 104.81 | 120.64 | 93.75 |
|  | 130.17 | 102.25 | 78.86 | 94.31 |
|  | 82.24 | 103.08 | 90.14 | 95.67 |
